# Supplementary figures and images for: Characterization of the endogenous DAF-12 ligand and its use as an anthelmintic agent in Strongyloides stercoralis
Source: eLife. 2021 Dec 7;10:e73535. doi: 10.7554/eLife.73535 (PMC8651287; doi:10.7554/eLife.73535)

Figure 4-figure supplement 1. Full gel images for the expression of Ss-CYPs.

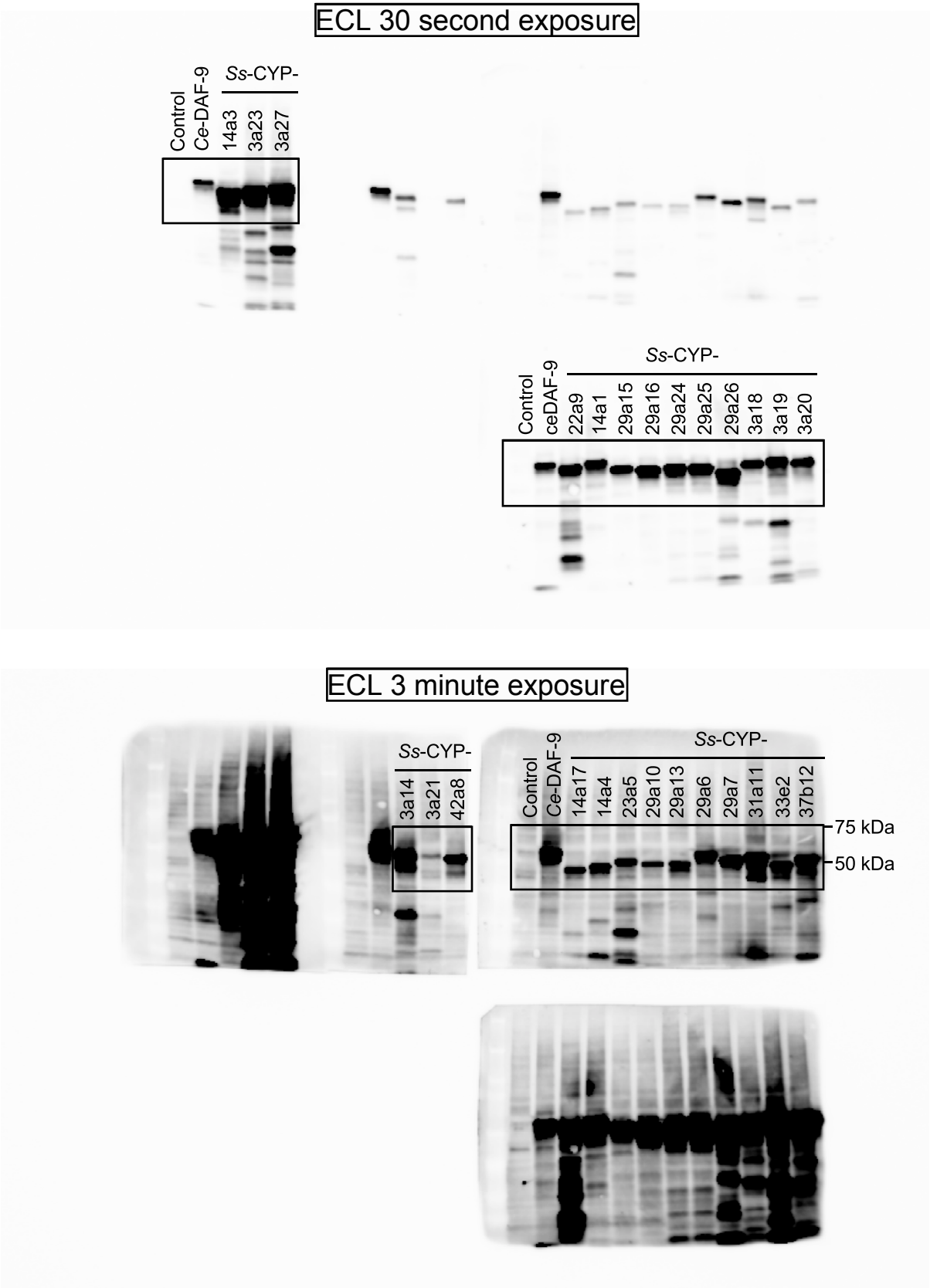

Supplement: Figure 4—figure supplement 1—source data 1. [file elife-73535-fig4-figsupp1-data1.pdf]

Figure 5B. Full gel images for single worm genotyping

2020-07-31 22a9 genotyping 2nd

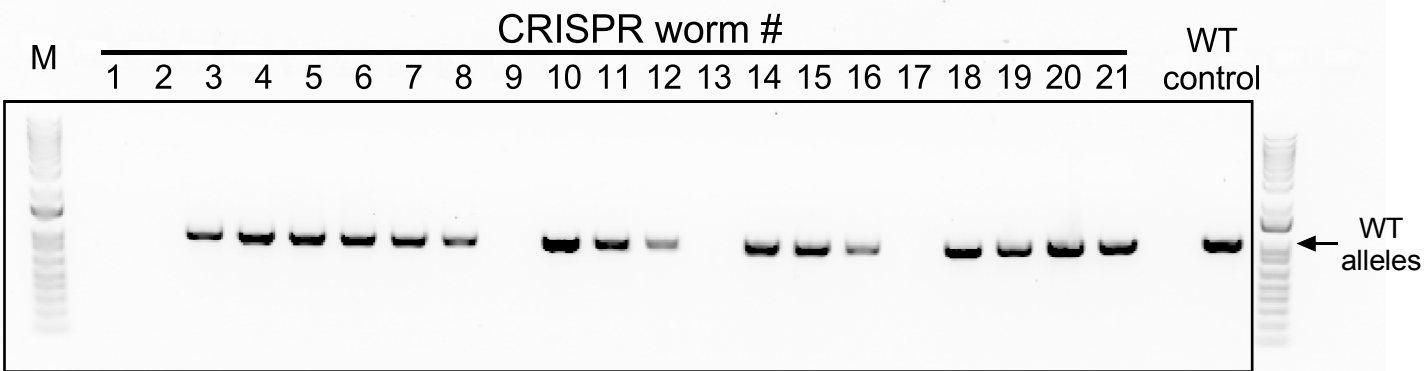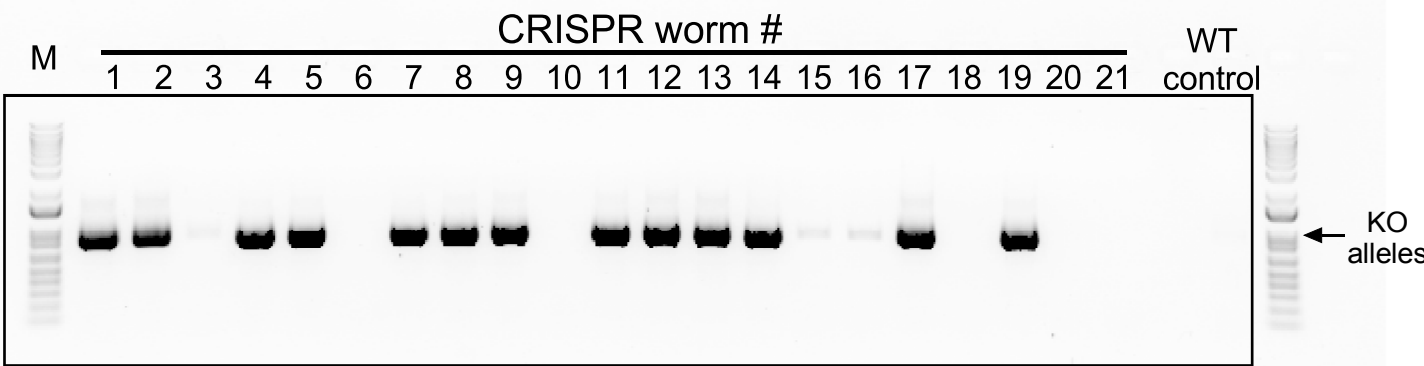

Supplement: Figure 5—source data 1. [file elife-73535-fig5-data1.pdf]
